# Association between adverse oral conditions and cognitive impairment: A literature review

**DOI:** 10.3389/fpubh.2023.1147026

**Published:** 2023-04-06

**Authors:** Tianhao Wei, Yifeng Du, Tingting Hou, Chunjuan Zhai, Yuqi Li, Wei Xiao, Keke Liu

**Affiliations:** ^1^Department of Neurology, Shandong Provincial Hospital Affiliated to Shandong First Medical University, Jinan, Shandong, China; ^2^Department of Neurology, Shandong Provincial Hospital, Cheeloo College of Medicine, Shandong University, Jinan, Shandong, China; ^3^Department of Cardiology, Shandong Provincial Hospital, Cheeloo College of Medicine, Shandong University, Jinan, Shandong, China; ^4^Department of Infection Control, Shandong Provincial Hospital Affiliated to Shandong First Medical University, Jinan, Shandong, China

**Keywords:** cognitive dysfunction, periodontitis, tooth loss, oral microflora, oral dysfunction

## Abstract

Oral environment deterioration results from a lack of self-cleaning ability in patients with cognitive dysfunction but is also a risk factor for cognitive dysfunction. Adverse oral conditions can be alleviated and improved through a self-management and medical examination. In this review, the epidemiological evidence of previous studies is integrated to highlight the relationship between periodontitis, tooth loss, oral flora, oral dysfunction and cognitive dysfunction, emphasizing the importance of oral health for cognition. The results show that poor oral condition is associated with cognitive impairment. Although many previous studies have been conducted, there is a lack of higher-level research evidence, different judgment criteria, and conflicting research results. There is a bidirectional relationship between oral health and cognitive dysfunction. A comprehensive analysis of the relationship between oral health and cognitive dysfunction that explores the relationship and takes measures to prevent cognitive dysfunction and control the progression of such diseases is warranted in the future.

## 1. Introduction

Cognitive dysfunction manifests mainly as impaired memory, thinking, calculation, judgment, language, and other abilities, which seriously affect the normal life of patients. Cognitive dysfunction is a continuously developing process, from cognitive decline to mild cognitive impairment (MCI) to dementia. With the increase in global aging, dementia has gradually become a serious global public health problem. Compared with developed countries such as Europe and North America, the incidence of dementia continues to increase in low-income countries such as Asia and Africa (1). According to the World Health Organization, there are approximately 10 million new patients with dementia worldwide every year, and the number is expected to reach 82 million by 2030, with a cost of up to 2 trillion dollars (2). This will pose huge challenges for national healthcare and social welfare systems. Alzheimer’s disease (AD) accounts for 60–80% of all dementia types (3); however, there are no effective treatments for any type of dementia to date, including AD and MCI. The main treatment method for patients with dementia involves symptomatic relief and access to necessary care and services to maximize the quality of life. Therefore, current research has focused on discovering risk factors for cognitive dysfunction in advance and taking effective and reasonable intervention measures to effectively reduce and delay the occurrence of dementia.

The oral cavity is an important part of the human body and an essential organ for maintaining a normal life and normal social communication. In recent years, numerous studies have shown significant associations between the oral cavity and various systemic diseases (cirrhosis, diabetes, sepsis, arthritis, and atherosclerosis) (4, 5). In addition, other studies have shown that the oral cavity is related to nervous system diseases, and oral problems have a bidirectional correlation with cognitive dysfunction. Poor oral condition is a risk factor for cognitive dysfunction; in turn, poor cognition aggravates the deterioration of oral function (6). The correlation between oral problems and cognitive dysfunction has become a global research concern. In this review, we summarize the epidemiological studies on the effects of periodontal disease, tooth loss, oral flora, and oral function on cognitive dysfunction, providing scientific evidence for further oral and cognitive research.

## 2. Search strategy

We searched PubMed for articles published between 2000 and 2022 using combinations of the following terms: “Periodontitis,” “Periodontal disease,” “Tooth loss,” “Oral bacteria,” “Oral microbiome,” “Oral microbiota,” “Oral function,” “Dental occlusion,” “Dementia,” “Alzheimer’s disease,” “Mild cognitive impairment,” “Cognitive impairment,” and “Cognitive function.” Only articles published in English were considered.

The inclusion criteria were as follows: (1) cohort study or case–control study; (2) animal experiments and functional mechanism studies; (3) the main oral factors studied were periodontitis, tooth loss, oral flora, and oral dysfunction; (4) the article is in English.

**Figure 1:**
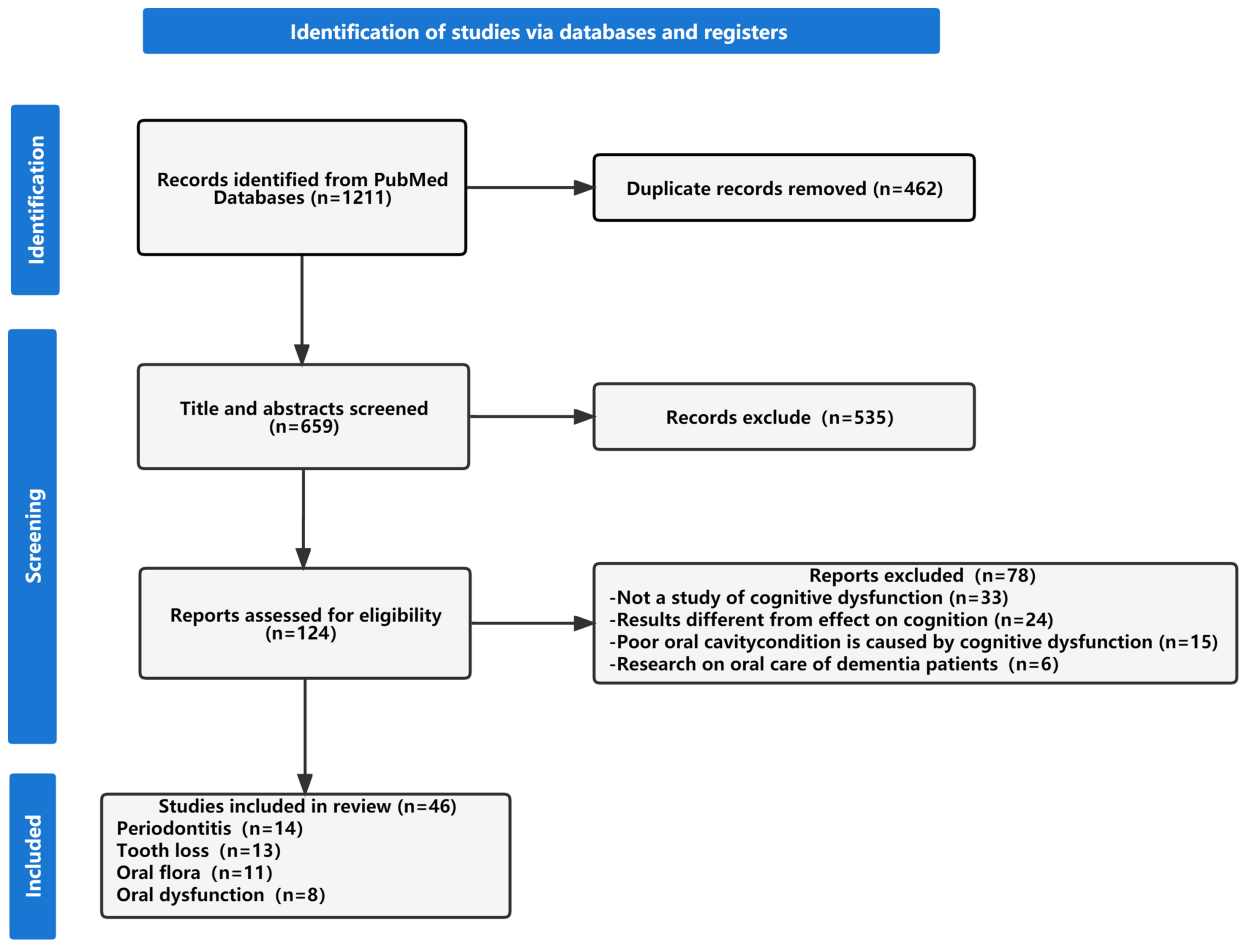
Flow chart of the literature search and study selection process.

In total, 1,211 articles were retrieved, and 659 articles remained after removing duplicates. The titles and abstracts were screened, and 170 articles remained after 489 articles that did not meet the title, and abstract criteria were excluded. Finally, another 124 articles were further excluded, and the final 46 articles were included in this review. Figure 1 shows a flowchart of the research article selection process.

## 3. Periodontitis and cognitive dysfunction

Periodontitis is a common dental disease that causes chronic inflammation of periodontal tissues through oral bacteria activity, which eventually leads to tissue destruction and tooth loss. Besides common dental inflammation, periodontitis can also induce chronic systemic inflammation leading to a serum proinflammatory response affecting neurological function and increasing the risk of cognitive impairment (7, 8). This manifests in the body as an increase in C-reactive protein and pro-inflammatory cytokine α in the blood and a decrease in anti-inflammatory markers (interleukin-10) (9). When the body is in a pro-inflammatory state for a long time, it can cause the expression of tight junctions that maintain the integrity of the blood–brain barrier to be reduced or misallocated, resulting in the interruption of the blood–brain barrier. At the same time, inflammatory factors have toxic effects on endothelial cells, leading to cell apoptosis and increasing the blood–brain barrier permeability. The presence of inflammatory factors can also increase blood–brain barrier permeability by activating microglia or stimulating astrocytes to secrete vascular endothelial growth factor-A (10). Due to the blood–brain barrier increased permeability and loss of complete protective function, inflammatory factors or endotoxins can penetrate the nervous system and eventually have an impact on brain function (11).

In a cross-sectional study of periodontitis and cognitive dysfunction published in 2008, Yu et al. found that periodontitis was more common in participants with low cognitive function scores (12). In recent years, some studies have also found that periodontitis is related to early cognitive dysfunction and AD. People with cognitive dysfunction have worse oral health and a higher incidence of periodontal diseases, such as sparse root tips, deepening periodontal pockets, and dental caries (13). In addition, several epidemiological studies have also shown a significant link between periodontitis and cognitive dysfunction. Noble et al. determined associations with cognitive function by measuring serum markers of periodontitis (14). They showed that individuals with higher *Porphyromonas gingivalis* immunoglobulin G levels had a higher rate of impaired performance in verbal memory and subdivision tests. This association persisted even after adjusting for confounding factors, such as social population and underlying diseases. In a Danish study, Kamer et al. used the digit symbol and block design tests to assess participants’ cognitive function (15). Compared with participants without periodontitis, those with periodontitis had significantly lower digit symbol test scores (*p* = 0.02). A Japanese study analyzed the correlation between periodontal disease and cognitive impairment in an Asian population, and the European Consensus criteria for the periodontal disease were used to determine periodontal conditions and the Mini-Mental State Examination (MMSE) and Hasegawa Dementia Scale-Revised criteria were used to determine cognitive outcome variables (16). Periodontitis and cognitive score were still significantly correlated after adjusting for all co-variables. The former score was 2.21 (1.01–4.84, *p* < 0.05), and the latter score was 4.85 (1.29–18.15, *p* < 0.05). In a prospective cohort study of over 6,000 participants, Lee et al. found that periodontitis patients had a significantly higher risk of developing dementia than healthy controls. The risk increased after adjusting for sociodemographic characteristics (OR = 1.16, 95% CI = 1.01–1.32) (17). Chen et al. conducted a large retrospective cohort study including 27,963 people >50 years to determine the relationship between periodontitis and AD (18). Patients who had periodontitis for more than 10 years had more than a 1.7-fold risk of developing AD, which showed that long-term exposure to periodontitis has a significant effect on brain cognitive function. A large cross-sectional study by Sung et al. used Neurobehavioral Evaluation System 2 to judge cognitive function in participants and showed that severe periodontitis led to worse cognitive status, and the correlation remained significant after adjusting for confounding factors (19). Iwasaki et al. followed 179 participants for 5 years using two different definitions from the Periodontology Group and the American Academy of Periodontology to assess periodontitis severity (20). In this study, periodontitis severity was associated with cognitive impairment regardless of the standard definition.

In summary, studies have shown a correlation between periodontitis and cognitive dysfunction; to a certain extent, periodontitis is also a risk factor for cognitive impairment. However, most of the above studies were cross-sectional, so the causal relationship is difficult to explain. Although some were cohort studies, the cognitive judgment and diagnostic criteria for periodontitis may vary among them, which will inevitably lead to different results. In addition, the onset of dementia involves multiple mechanisms, and few studies have taken into account important confounding factors, such as family history, the susceptibility gene apolipoprotein E, nutrition level, and education level. In addition, periodontitis severity may also have different effects on cognition, but only one study considered the relationship between periodontitis severity and cognition (20). Therefore, this association requires further study. At present, there are few cohort studies in the field of periodontitis, and further large-population studies with the same diagnostic criteria are needed to clarify the mechanism of action linking periodontitis and cognitive dysfunction.

## 4. Tooth loss and cognitive dysfunction

Tooth loss is a common oral disease that is more common in the elderly population and is associated with age, smoking, economic status, poor diet, and various oral pathological factors (21). Evidence shows that tooth loss is related to oral health and cognitive function. A lack of nutrients, such as B vitamins, may have an impact on cognition due to changes in eating habits caused by missing teeth (22, 23). Animal experiments have confirmed that prolonged molar deprivation can reduce the expression levels of brain-derived neurotrophic factor (BDNF), which is related to hippocampal learning and memory. Decreased BDNF expression is also present in patients with AD, and the number of hippocampal vertebral cells is decreased, leading to cognitive dysfunction (24, 25). In imaging studies, patients with tooth loss showed significant gray matter shrinkage in brain areas responsible for memory and cognition, such as the hippocampus, caudate nucleus, and temporal pole (26). In addition, tooth loss was associated with decreased total gray matter volume in the brain (27), suggesting that tooth loss increases the risk of shrinking brain regions associated with memory, learning, and cognition.

A prospective study including 597 older American men showed that each tooth lost per decade increased the risk of MMSE score reduction by 9–12% over 32 years of follow-up (28). In a 5-year follow-up study of 11,140 patients with type 2 diabetes, Batty et al. found that participants with no teeth had a significantly higher risk of dementia and cognitive decline than those with 1–22 or more teeth (29). In a cross-sectional study of 3,063 participants, patients with dementia averaged 18.7 missing teeth, compared with 11.8 and 9.3 for patients with MCI and normal cognition, respectively, and >16 lost teeth were significantly associated with dementia (OR: 1.5, 95% CI: 1.12–2.18), indicating that tooth number is associated with cognitive function (30). In a longitudinal cohort study of aging in the United Kingdom, participant memory was assessed using the number of words they could recall. Patients without teeth had worse memory, recalling 0.88 fewer words than patients with teeth, as well as worse motor ability (31). The association between memory and motor ability differed significantly by age, and it was more significant in the elderly aged 60–74 years. A 13-year longitudinal study in China conducted by Li et al. found that cognitive function gradually declined with time but retained a significant correlation with the number of teeth (32). Evidently, the MMSE score decreased by 0.01 points for each missing tooth, and the number of teeth significantly correlated with the time passage. This suggests that older adults with more teeth have a better cognitive function and a slower rate of cognitive decline. A 5-year Japanese cohort study analyzed the relationship between the number of remaining teeth and all-cause dementia, AD, and vascular dementia (33). The study showed a significant association between all-cause dementia and tooth number, which was preserved after model adjustment. The number of remaining teeth was negatively associated with the risk of AD, but this association was not statistically significant after model adjustment. A prospective cohort study also partially confirmed the association between tooth loss and higher risk of dementia. In a cross-sectional study, Kato et al. (34) evaluated cognitive diagnosis in elderly Japanese communities and showed that MMSE scores decreased along with the number of teeth. In addition, participants with >20 total teeth (including natural teeth and dentures) had significantly higher MMSE scores than those with <19 total teeth, suggesting that denture use may exert a protective effect on cognitive function.

In conclusion, the above studies show a significant association between tooth loss and cognitive dysfunction, which has been verified in longitudinal studies in Asian, European, and American populations. Two cross-sectional studies found similar results, where tooth loss indicated poorer cognitive function. Some studies use MMSE as a diagnostic assessment tool for cognition; however, considering its limitations, it can be combined with better diagnostic criteria to obtain more accurate results. In addition, cognitive diagnostic criteria are worth considering, as the diagnosis workload will inevitably increase due to the large number of patients involved and the time-consuming nature of longitudinal cohort studies. Moreover, different studies have used different criteria to count the number of teeth. In their study, Li et al. used the number of self-reported teeth as the standard, and subsequent data cleaning and examination were carried out, which inevitably increased the workload and greatly reduced data reliability (32). Cohort studies by Batty et al. (29) and Takeuchi et al. (33) counted “complete/partial attachment to the gums as a tooth” and “healthy, treated or repaired teeth,” respectively, but the mechanism by which teeth affect cognitive function puts more emphasis on chewing. Therefore, it is worth studying the criterion of “complete, healthy, functional teeth after treatment or repair.” In addition, the effect of dentures on cognitive function has rarely been mentioned in cohort studies, and their long-term protective effect still requires further demonstration in large, high-quality epidemiological studies.

## 5. Oral flora and cognitive dysfunction

Oral cavity is a gathering place for many microorganisms, whose balance is essential for maintaining overall health. Oral microbial imbalance is the main cause of various oral diseases and an important risk factor for cardiovascular, digestive, and nervous system diseases (5). Some studies have found the presence of *Porphyromonas gingivalis* and *Treponema* in brain tissue, trigeminal ganglion, and cortex samples of patients with AD (35, 36). Therefore, oral bacteria appear to be involved in the development of cognitive dysfunction. Human immunity and saliva are crucial for maintaining oral health and regulating the balance of oral flora. However, with age increase, saliva secretion decreases, and immune function weakens, reducing the body’s ability to inhibit oral flora overgrowth and non-oral species invasion (37, 38), eventually leading to a pro-inflammatory response and weakening the protective effect of the blood–brain barrier. The spread of bacteria to the brain impacts brain function. In addition, oral bacteria can produce toxins such as lipopolysaccharides, arg-gingipain, and lys-gingipain, which damage the tau protein, which is responsible for neuronal function (39). Oral bacteria can also cross the blood–brain barrier and cause transient encephalitis, which leads to short-term memory impairment. Meanwhile, the persistent infection produces lasting cognitive damage in the brain (40).

Liu et al. used 16S rRNA sequencing to analyze the differences between the saliva microflora of patients with AD and healthy people (41). The variety and richness of saliva microflora from patients with AD were significantly lower than those of healthy controls, and the abundance of *Moraxella*, *Leptotrichia micronomyces*, and *Sphaerochaeta* in patients with AD increased, while *Rhodotella* abundance decreased. In addition, salivary flora diversity decreased in patients with AD, the bacterial community was disturbed, and it invaded the brain to affect neurological function. Yang et al. collected oral samples from 68 patients with MCI and a control group, analyzed the characteristics of oral microorganisms and explored their association with MCI inflammation markers (42). There was no difference in alpha- and beta-diversity between the two groups and no change in oral microflora. Several inflammatory factors related to cognitive function, such as matrix metallopeptidase 10 and chemokines in the cerebrospinal fluid of the MCI group, were different from those of the control group, and there was a correlation between the oral microbiome and inflammation. Wu et al. (43) analyzed the bacteria in the dental plaque of patients with AD and a control group and used alpha diversity to assess the difference between groups. Oral microflora diversity in the AD group was lower than that in the control group, and the number of *Lactobacillus*, *Streptococcus*, and *Bacteroides* increased significantly in patients with AD, while the number of *Clostridium* decreased significantly. Holmer et al. collected 95 subgingival specimens to identify oral microflora in three case groups (AD, MCI, and subjective cognitive dysfunction). The microbial alpha diversity of subjective cognitive dysfunction was significantly higher than that of the control group, and the microflora of the MCI group was particularly rich and diverse. Compared with non-dementia patients, the oral microbiota showed consistent changes and was significantly associated with periodontal disease in three case groups (44). A Canadian case–control study with 90 participants collected oral specimens from the salivary glands on both sides of the mouth and under the tongue, also using 16S microbial sequencing technology (45). Contrary to Wu et al. (43), this study showed higher alpha diversity of oral microorganisms and decreased *Streptococcus* and *Actinomyces* abundance in patients with AD, compared with controls.

In conclusion, although studies have shown no significant difference in oral flora between patients with MCI and healthy participants, oral microbes are associated with inflammatory factors (42), and the MCI group showed a high degree of diversity in a subsequent study (44). MCI is the early stage of dementia, whose development can be effectively controlled by detecting risk factors and adopting countermeasures. At present, there are few research studies in this field, and more large-cohort studies are needed to verify the current results. Moreover, four studies have analyzed the oral microbiota of patients with AD, two of which showed reduced diversity. Two of the low-diversity studies included Chinese people (Asian), and the other two included Swedes and Canadians (Caucasian), so it is worth exploring the impact of race or dietary differences. Moreover, confounding factors, such as daily habits, socioeconomic conditions, and drug use, were not taken into account in the study. Oral specimens were also collected from different sites in different studies, and the number of participants was small, which may also impact the results. For pathogenic bacteria, the distance from the mouth to the brain is shorter than that from other organs. Studies have shown that oral bacteria can penetrate the blood–brain barrier and affect brain neurological functions by causing neuroinflammation through soluble surface proteins or the production of lipopolysaccharides, exotoxins, and other substances (39, 46). Therefore, an in-depth exploration of the relationship between oral microbes and cognitive dysfunction may provide a feasible method to reduce the risk of cognitive dysfunction.

## 6. Oral and cognitive dysfunction

Commonly, oral dysfunction includes decreased chewing ability, decreased tongue motor ability, decreased tongue pressure, subjective eating, and swallowing difficulties (47). These problems can be summarized as “oral fragility,” a term introduced by Japan’s Ministry of Health, Labor, and Welfare in 2013 to emphasize the role of oral function in overall health. Evidence suggests that oral fragility significantly increases the risk of frailty, sarcopenia, disability, and death in older adults and is associated with cognitive impairment (4, 48).

Some studies have found that chewing can increase blood perfusion and stimulate neural activity in the brain (4), exerting a positive impact on memory and improving cognitive ability (49). On the contrary, declining chewing function may negatively impact brain function and cause cognitive dysfunction (50). In a cross-sectional study of 502 participants, Cardoso et al. determined the number of functional masticatory units in participants through visual examination and analyzed its association with cognitive function (51). A positive correlation was seen between the number of functional masticatory units and the Mini Cognitive Examination score, as more chewing units indicated better cognitive function. Han et al. achieved similar results in a longitudinal cohort study involving 411 participants (52), where more functional teeth and occlusal units indicated a lower probability of cognitive decline. These two studies provide strong epidemiological evidence for an association between chewing function and cognitive dysfunction.

A cross-sectional study on oral and cognitive function involving 1,118 people in Japan revealed that tongue pressure and oral diadochokinesis were significantly associated with MMSE scores after adjusting for relevant factors (53). Moreover, pathway analysis showed that tongue pressure was related to decreasing MMSE scores, and it affected cognitive function through oral diadochokinesis. Egashira et al. used the Japanese version of the Montreal Cognitive Assessment Form to assess cognitive status in a 50-person dental outpatient cross-sectional study, showing that tongue pressure and tooth count were significantly lower in the cognitively declining group than in the healthy group (54). A cross-sectional study by Suzuki et al. showed that the maximum bite force of patients with cognitive dysfunction was significantly reduced, and a high proportion of patients had tongue and lip dysfunction (55). After adjusting for gender and age, bite force was still correlated with cognitive function.

The above research shows that traditional oral diseases, such as periodontitis and tooth loss, are associated with cognitive dysfunction, as is oral dysfunction. Epidemiological studies have shown an association between reduced chewing function and cognitive impairment, but the accuracy of their results is questionable, given that assessments were based on direct oral examination, and they did not take into account socioeconomic levels, malnutrition caused by mastication, and other factors associated with cognitive impairment. The cross-sectional design used in studies of bite force, tongue pressure, oral coherent movement, and cognitive function could not establish causality and included a small number of participants. In all studies, scales were used to judge cognitive function. However, in an ideal situation, neuropsychological tests and imaging results should be used to diagnose cognition in each participant. Therefore, more longitudinal cohort studies in large populations are required to further clarify the correlation and causal relationship between oral function and cognitive dysfunction. Oral cavity is an important organ of the human body. Exploring the functional relationship between the oral cavity and brain organs may produce a new understanding of brain cognitive function. At present, most of the studies on oral function have been conducted in Asian populations. To further understand the relationship between oral function and cognitive function, besides longitudinal studies in large populations, different regions should be discussed to obtain more convincing results.

## 7. Conclusion

**Figure 2:**
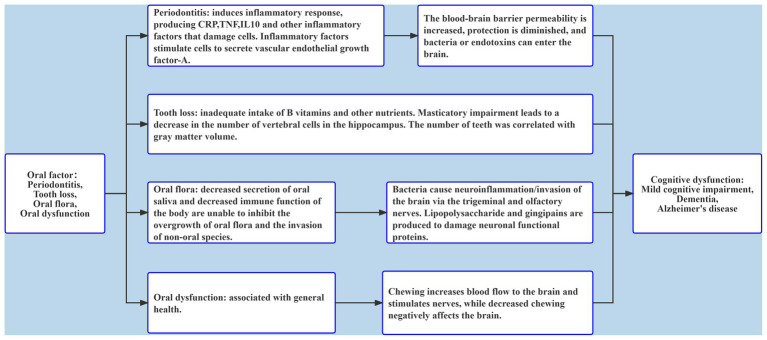
The role of oral factors including periodontitis, tooth loss, oral flora, and oral dysfunction in cognitive dysfunction.

This review focused mainly on the relationship between periodontitis, tooth loss, oral flora, oral dysfunction, and cognitive dysfunction, and its results showed that adverse oral conditions would greatly impact patient cognitive function (Figure 2). Most studies on periodontitis and tooth loss used different judgment criteria for oral and cognitive status, and the included confounding factors were not comprehensive. Oral flora has a great impact on cognitive function, but the results of changes in oral flora in patients with cognitive impairment are conflicting. At present, there are few studies on oral dysfunction and cognition in which the study population is relatively concentrated and oral function is diverse. Longitudinal studies with large populations can better clarify the association. Moreover, most of the existing studies have analyzed a single adverse oral condition, so it was necessary to try to analyze the association between multiple adverse oral factors and cognition in the same population, which helps find the association and take timely measures. To a large extent, poor oral health can be improved or treated with current medical technology. Therefore, it is of great significance to comprehensively explore the association between oral health status and cognitive dysfunction for the prevention or early detection of risk factors for cognitive dysfunction. However, one limitation of this review was that different studies included no uniform definition for cognitive dysfunction and oral conditions, as well as many influencing factors. Therefore, the final research results should be observed with caution. In future studies, unified standards should be adopted, research methods should be carefully designed, more rigorous tests should be conducted, and longitudinal cohort studies of large populations should be adopted more frequently to ensure representative results. Current medical technology can improve or treat adverse oral conditions to a large extent. Therefore, the relationship between oral problems and cognitive dysfunction is certainly invaluable to preventing and facilitating early detection of oral risk factors related to cognitive dysfunction.

## Author contributions

YD, WX, and KL contributed to the conception and design of the study. TW, TH, and YL organized the database. TW, WX, CZ, and KL wrote the manuscript. All authors contributed to the manuscript revision and read and approved the submitted version.

## Funding

This work was supported by grants from the Clinical Medicine Technology Innovation Program (grant no. 202019080), the Natural Science Foundation of Shandong Province (grant no. ZR2022QH106), the National Natural Science Foundation of China (grant nos. 81861138008 and 81772448, 82011530139, and 82171175), the National Key R&D Program of China Ministry of Sciences and Technology (grant nos. 2017YFC1310100 and 2022YFC3501404), the Academic Promotion Program of Shandong First Medical University (grant no. 2019QL020), the Integrated Traditional Chinese and Western Medicine Program in Shandong Province (grant no. YXH2019ZXY008), and the Brain Science and Brain-Like Intelligence Technology Research Projects of China (grant nos. 2021ZD0201801 and 2021ZD0201808), and Shandong Provincial Key Research and Development Program (grant no. 2021LCZX03). The funding agencies had no role in the study design, data collection and analysis, the writing of this article, and in the decision to submit the work for publication.

## Conflict of interest

The authors declare that the research was conducted in the absence of any commercial or financial relationships that could be construed as a potential conflict of interest.

## Publisher’s note

All claims expressed in this article are solely those of the authors and do not necessarily represent those of their affiliated organizations, or those of the publisher, the editors and the reviewers. Any product that may be evaluated in this article, or claim that may be made by its manufacturer, is not guaranteed or endorsed by the publisher.
